# Effect of zinc supplementation on growth Hormone Insulin growth factor axis in short Egyptian children with zinc deficiency

**DOI:** 10.1186/1824-7288-38-21

**Published:** 2012-05-24

**Authors:** Rasha T Hamza, Amira I Hamed, Mahmoud T Sallam

**Affiliations:** 1 Department of Pediatrics, Ain Shams University, 36 Hisham Labib street, off Makram Ebeid street, Nasr City, Cairo, Egypt; 2 Department of Clinical Pathology, Faculty of Medicine, Ain Shams University, Cairo, Egypt; 3 Department of Clinical and Chemical Pathology, National Research Center, Cairo, Egypt

**Keywords:** Egyptian, Growth hormone, Insulin growth factor-1, Zinc

## Abstract

**Background:**

The relationship between zinc (Zn) and growth hormone-insulin growth factor (GH-IGF) system and how Zn therapy stimulates growth in children has not been clearly defined in humans. Thus, we aimed to assess GH-IGF axis in short children with Zn deficiency and to investigate the effect of Zn supplementation on these parameters.

**Methods:**

Fifty pre-pubertal Egyptian children with short stature and Zn deficiency were compared to 50 age-, sex-, and pubertal stage- matched controls. All subjects were subjected to history, auxological assessment and measurement of serum Zn, IGF-1, insulin growth factor binding protein-3 (IGFBP-3); and basal and stimulated GH before and 3 months after Zn supplementation (50 mg/day).

**Results:**

After 3 months of Zn supplementation in Zn-deficient patients, there were significant increases in height standard deviation score (SDS, P = 0.033), serum Zn (P < 0.001), IGF-1 (P < 0.01), IGF-1 standard deviation score (SDS,P < 0.01) and IGFBP-3 (P = 0.042). Zn rose in all patients but reached normal ranges in 64 %, IGF-1 levels rose in 60 % but reached normal ranges in 40 % and IGFBP-3 levels rose in 40 % but reached reference ranges in 22 %. Growth velocity (GV) SDS did not differ between cases and controls (p = 0.15) but was higher in GH-deficient patients than non-deficient ones, both having Zn deficiency (p = 0.03).

**Conclusion:**

Serum IGF-1 and IGFBP-3 levels were low in short children with Zn deficiency, and increased after Zn supplementation for 3 months but their levels were still lower than the normal reference ranges in most children; therefore, Zn supplementation may be necessary for longer periods.

## Introduction

Although Zn deficiency is a common nutritional problem around the world, especially in children in developing countries where diets have less Zn available, it is difficult to identify this problem. Zn is important for the metabolic activity of more than two hundred enzymes. It is essential for cell replication, and deoxyribonucleic acid (DNA) and protein synthesis. Zn deficiency is reported to be associated with impairment of growth, testicular functions, appetite, and sense of taste, delay in wound healing, immune resistance and memory. Zn deficiency interferes with the metabolism of thyroid hormones, androgens and growth hormone (GH). It is not known what mechanism is responsible for growth retardation in Zn deficiency and how Zn therapy stimulates growth in children [1].

The principal regulator of growth in the body is the GH-IGF system [2]. It appears that Zn supplementation has positive effects on growth and IGF-1 levels in various groups of Zn-deficient children. However, the exact mechanism of Zn deficiency and the mechanism by which Zn supplementation affects GH secretion and IGF-1 levels is not well delineated. Furthermore, the effect of Zn supplementation on the GH-IGF axis in non Zn-deficient short children is not known [3]. Some studies have reported positive effects of Zn supplementation on growth in various groups of Zn-deficient children [3-5]. However, in some studies, this effect was not observed [6,7].

With this background, we were stimulated to assess GH-IGF axis in short children with Zn deficiency and to investigate the effect of Zn supplementation on these parameters.

## Subjects and methods

### Study population

This prospective case control study was conducted on 50 pre-pubertal Egyptian children with short stature and Zn deficiency [27 males (54 %), 23 females (46 %)] whose ages ranged between 3.2 and 10.9 years (mean age: 6.5 ± 3.05 years). All patients were shorter than expected for their chronological age and sex (height SDS < −2) [8]. Patients were recruited from Nile Hospital, Egyptian Health Insurance (n = 32) and the Pediatrics Outpatient Clinic, Children's hospital, Faculty of Medicine, Ain Shams University, Cairo, Egypt (n = 18) during the period from the beginning of April 2010 to the end of August 2011. Cases were compared to 50 healthy age-, sex- and pubertal stage- matched children serving as controls who had normal serum Zn levels. They were 28 males (56 %) and 22 females (44 %) whose ages ranged between 3.5 and 10.8 years (mean age: 6.7 ± 2.05 years). They were recruited from the Pediatrics Outpatient clinic, Children`s hospital, Faculty of Medicine, Ain shams University, Cairo, Egypt.

Children with chronic systemic diseases, bone dysplasias, dysmorphic syndromes, chronic malabsorption, other nutritional deficiencies, short stature with normal serum Zn levels (>109 μg/dl), other endocrine problems e.g hypothyroidism and history of long-term medications that might influence the GH-IGF axis were excluded from the study. In addition, all patients were not on GH therapy during the time of the study.

An informed written consent of participation in the study was signed by the parents or legal guardians of the studied subjects. This study was approved by the Bioethical Research Committee, Faculty of Medicine, Ain Shams University hospitals, Cairo, Egypt.

### Study measurements

All studied children were subjected to:

· Medical history and clinical assessment: laying stress on therapeutic history, systemic examination and dysmorphic features.

· Auxological parameters: Height was measured to the nearest 1.0 mm with a Harpenden wall mounted stadiometer and weight to the nearest 0.1 kg on electronic scales together with calculation of height for age, weight for height and height velocity Standard Deviation Scores (SDSs) [8]. Body Mass Index (BMI) was calculated using the formula weight (in kg)/height^2^ (in meters) together with calculation of BMI SDS calculated from the age- and sex-specific reference values [9].

· Tanner pubertal staging: for assessment of pubertal status according to the standards of Tanner and Whitehouse (1976) [10]

· Laboratory assays: All blood samples were taken in the morning after an overnight fast for measurement of the following:

Serum Zn which was measured by a spectrophotometric method after deproteinization of samples. The normal reference range ranged from109-167 ng/dl while serum Zn values below 109 μg/dl indicated Zn deficiency [11].

Serum IGF-1 and IGFBP-3 that were measured using a solid phase, enzyme labeled chemiluminescent immunometric assay. Values were expressed as absolute values (ng/ml) and were also expressed as SDS for age and sex according to normal reference ranges of Elmlinger et al., 2004 [12]

Basal and stimulated serum GH levels after insulin induced hypoglycemia, in which baseline GH and glucose samples were withdrawn and regular insulin was administered in a dose of 0.1 unit/kg intravenously and samples for measurement of GH and glucose were withdrawn at 30, 60, 90 and 120 minutes. Blood glucose must decrease by 50 % of the initial value or to <40 mg%. In addition, GH provocation test by clonidine was done to confirm the diagnosis of growth hormone deficiency (GHD) in which a baseline GH sample was withdrawn and clonidine was administered in a dose of 5 μ**g**/kg orally and samples for measurement of GH were withdrawn at 30, 60, 90 and 120 minutes. Normally, GH should rise to a peak of ≥10 ng/ml at any of the post-stimulatory samples. Patients with peak GH levels <10 ng/ml by the 2 stimulatory tests were considered GH-deficient [13]. Serum GH concentrations were measured using commercial reagents (Pharmacia Diagnostics, Uppsala, Sweden) by a solid-phase, enzyme-labeled chemiluminiscent immunometric assay (by the Immulite, 2000 Analyzer, Siemens).

· Bone age (BA) was done by performing plain X ray left hand and wrist together with calculation of bone age SDS according to the standards of Greulich and Pyle [14].

· Oral Zn sulfate (50 mg/day elemental Zn) was given to all subjects for 3 months after which auxological assessment was repeated and serum samples were withdrawn to repeat all laboratory assays. In addition, growth velocity (GV) after 3 months of Zn therapy was calculated. GV is the variable that describes the patient's one year or 6 months or 3 months height velocity. GV SDS was calculated according to the norms of Tanner etal, 1966 [8].

### Statistical analysis

The results were analyzed using the Statistical Package for the Social Science (SPSS) version number 10, Echosoft corp; USA, 2005. Description of quantitative variables was in the form of mean ± standard deviation and range while that of qualitative variables was in the form of frequency and percentage. Student’s t-test of 2 independent samples was used to compare 2 quantitative variables while Wilcoxon Signed Rank test was used to compare 2 dependent groups before and after Zn supplementation. Spearman correlation coefficient test (r-test) was used to rank different variables against each other either directly or indirectly. A p-value of <0.05 was considered significant.

## Results

### Characteristics of studied cases

Descriptive clinical, laboratory and radiological data of studied cases are presented in Table 1. Of the 50 studied cases, serum IGF-1 levels were below the normal age- matched reference ranges in 47(94 %) while serum IGFBP-3 concentrations were below the normal age- matched reference ranges in all children (100 %). In addition, GHD was diagnosed in 25 cases (50 %), that is, a peak GH of < 10 ng/ml by 2 GH provocation tests. The mean GV of all studied cases after 3 months of Zn supplementation was 0.55 ± 0.1 (range:0.1-2.23) with a GV SDS of +1.01 ± 0.2 (range: -0.5 - +2.11, Table 1).

**Table 1:** Descriptive clinical, laboratory and radiological data of studied cases (n = 50)

	Mean ± SD	Range
Age (years)	6.5 ± 3.05	3.20-10.90
Height SDS	-3.12 ± 0.2	-2.15- -4.21
-GV after Zn (cm/3 months)	0.55 ± 0.1	0.1-2.23
-GV SDS after Zn	+1.01 ± 0.2	-0.5 - +2.11
- Weight SDS	-1.88 ± 0.62	-2.01- +0.55
- BMI SDS	-1.77 ± 0.41	-2.10- -0.78
- Serum Zn (μg/dl)	56.76 ± 7.90	37.52-93.11
-Peak GH after insulin stimulation (ng/ml)	10.65 ± 2.01	7.61-13.20
-Peak GH after clonidine stimulation (ng/ml)	11.14 ± 1.41	6.92-14.11
- IGF-1 (ng/ml)	96.72 ± 11.5	36.52-115.69
-IGF-1 SDS	-2.46 ± 0.11	-3.22- +0.55
- IGFBP-3 (ng/ml)	2436.12 ± 392	1195.45-3898.15
-BA (years)	3.7 ± 1.05	1.81-9.25
-BA SDS	-1.75 ± 1.14	-2.44 - +0.22

Results are expressed as mean ± SD and range, Zn: zinc, SDS: Standard deviation score, GV: Growth velocity, BMI: Body mass index, GH: Growth hormone, IGF-1: Insulin like growth factor-1, IGFBP-3: Insulin like growth factor binding protein-3, BA: bone age.

### Cases versus controls

Height SDS (p < 0.001), serum Zn (p < 0.001), peak GH after insulin (P = 0.04) and clonidine stimulation (P = 0.041), IGF-1 (P < 0.001), IGF-1 SDS (p < 0.001), IGFBP-3 (P < 0.001), BA (p = 0.02) and BA SDS (p < 0.001) were significantly lower among cases than controls. On the other hand, other clinical parameters and; GV and GV SDS did not differ between both groups (p > 0.05, Table 2).

**Table 2:** Comparison of clinical, laboratory and radiological data among cases and controls

	Cases	Controls	t	p
	(N = 50)	(N = 50)		
Age (years)	6.5 ± 3.05	6.7 ± 2.05	1.2	0.11
	(3.2 -10.9)	(3.5-10.8)		
Sex , n(%)				
Males	27(54 %)	28 (56 %)	1.11	0.23
Females	23(46 %)	22(44 %)		
Height SDS	-3.12 ± 0.2	+1.04 ± 0.5	19.88	<0.001***
	(-2.15- -4.21)	(+0.99 - +1.65)		
GV after Zn (cm/3 months)	0.55 ± 0.1	0.61 ± 0.1	0.82	0.11
	(0.1-2.23)	(0.2-3.13)		
GV SDS	+1.01 ± 0.2	+1.21 ± 0.1	1.3	0.15
	(-0.5 - +2.11)	(-0.3 - +2.31)		
Weight SDS	-1.88 ± 0.62	-1.02 ± 0.71	1.7	0.2
	(-2.01 - +0.55)	(-1.20 - +1.45)		
BMI (kg/m2)	16.41 ± 1.56	17.51 ± 0.52	1.0	0.3
	(14.33-20.12)	(14.33-20.12)		
BMI SDS	-1.77 ± 0.41	-1.02 ± 0.31	1.99	0.082
	(-2.10- -0.78)	(-1.30 - +0.23)		
Serum Zn (μg/dl)	56.76 ± 7.90	136.70 ± 8.10	22.5	<0.001***
	(37.52-93.11)	(123.17-149.50)		
Peak GH after insulin stimulation (ng/ml)	10.65 ± 2.01	14.25 ± 3.01	6.50	0.04*
	(7.61-13.20)	(10.91-18.30)		
Peak GH after clonidine stimulation (ng/ml)	11.14 ± 1.41	15.24 ± 2.11	6.81	0.041*
	(6.92-14.11)	(11.12-19.21)		
IGF-1 (ng/ml)	96.72 ± 11.5	286.62 ± 12.5	12.86	<0.001***
	(36.52-115.69)	(198.72-325.19)		
IGF-1 SDS	-2.46 ± 0.11	+1.46 ± 0.21	17.66	<0.001***
	(-3.22- +0.55)	(+0.89 - +1.85)		
IGFBP-3 (ng/ml)	2436.12 ± 392	3936.22 ± 194	15.12	<0.001***
	(1195.45-3898.15)	(3721.45-4498.15)		
-BA (years)	3.7 ± 1.05	6.8 ± 1.6	6.11	0.020*
	(1.81-9.25)	(3.31-11.25)		
-BA SDS	-1.75 ± 1.14	+0.85 ± 1.20	13.24	<0.001***
	(-2.44 - +0.22)	(+1.04 - +0.22)		

Results are expressed as mean ± SD and range frequency and percentage, *p < 0.05, **p < 0.01, ***p < 0.001, GHD: growth hormone deficiency, SDS: Standard deviation score, GV: growth velocity, Zn: zinc, BMI: Body mass index, Zn: zinc, GH: Growth hormone, IGF-1: Insulin like growth factor-1, IGFBP-3: Insulin like growth factor binding protein-3, BA: bone age.

### Cases with GHD versus cases without GHD

Height SDS (p = 0.043), serum Zn (p < 0.001), peak GH after insulin (P = 0.021) and clonidine stimulation (P = 0.01), IGF-1 (P < 0.001), IGF-1 SDS (p < 0.001), IGFBP-3 (P = 0.01), BA (p = 0.03) and BA SDS (p = 0.01) were significantly lower among cases with GHD when compared to those without GHD. On the other hand, cases with GHD had significantly higher GV (p = 0.041) and GV SDS (p = 0.03) when compared to those without GHD (Table 3).

**Table 3:** Comparison of clinical, laboratory and radiological data of studied cases with and without GHD

	With GHD	Without GHD	t	p
	(N = 25)	(N = 25)		
Height SDS	−3.52 ± 0.3	−2.56 ± 0.1	6.11	0.043*
	(−2.85- -4.21)	(−2.15- -2.91)		
GV (cm/3 months)	1.21 ± 0.2	0.85 ± 0.1	5.99	0.041*
	(0.7-2.23)	(0.1-1.83)		
GV SDS	+1.56 ± 0.5	+0.91 ± 0.2	7.12	0.030*
	(+0.72 - +2.11)	(-0.5 - +1.86)		
Weight SDS	-1.68 ± 0.12	-1.58 ± 0.52	1.9	0.5
	(-1.99 - +0.55)	(-2.01- +0.25)		
BMI (kg/m2)	16.91 ± 0.25	17.31 ± 0.46	1.8	0.3
	(15.56-18.12)	(14.53-17.92)		
BMI SDS	-1.81 ± 0.10	-1.72 ± 0.32	2.1	0.5
	(-1.9- -0.78)	(-2.10- -0.82)		
Serum Zn (μg/dl)	45.71 ± 2.9	76.16 ± 5.9	18.12	<0.001***
	(37.52-61.55)	(39.12 - 93.11)		
Peak GH after insulin stimulation(ng/ml)	7.81 ± 0.1	11.61 ± 1.01	9.11	0.021*
	(7.61-8.91)	(10.55-13.20)		
Peak GH after clonidine stimulation (ng/ml)	7.14 ± 0.12	12.54 ± 1.21	10.16	0.01*
	(6.92-8.53)	(10.88-14.11)		
IGF-1 (ng/ml)	46.78 ± 6.5	100.12 ± 12.5	16.22	<0.001***
	(36.52-59.11)	(72.55-115.69)		
IGF-1 SDS	-2.72 ± 0.10	+0.38 ± 0.21	19.01	<0.001***
	(-3.22- -2.11)	(-1.20- +0.55)		
IGFBP-3 (ng/ml)	2006.22 ± 92	3445.11 ± 292	9.88	0.01*
	(1195.45 - 2111.26)	(3124.45 - 3898.15)		
-BA (years)	2.8 ± 1.0	5.7 ± 1.26	8.15	0.03*
	(1.81-5.9)	(4.-9.25)		
-BA SDS	-1.7 ± 0.12	-0.7 ± 0.14	11.22	0.01*
	(-2.44 - +0.01)	(-1.14 - +0.22)		

Results are expressed as mean ± SD and range, *p < 0.05, **p < 0.01, ***p < 0.001, GHD: growth hormone deficiency, SDS: Standard deviation score, GV: growth velocity, BMI: Body mass index, Zn: zinc, GH: Growth hormone, IGF-1: Insulin like growth factor-1, IGFBP-3: Insulin like growth factor binding protein-3, BA: bone age.

### Characteristics of cases before and 3 months after Zn supplementation

On comparing clinical, laboratory and radiological parameters among all studied patients before and 3 months after Zn supplementation, there were significant increases in height SDS (P = 0.033), serum Zn (P < 0.001), IGF-1 (P < 0.01), IGF-1 SDS (p < 0.01) and IGFBP-3 (P = 0.042) while other clinical and laboratory parameters did not differ 3 months after Zn supplementation (P > 0.05, Table 4).

**Table 4:** Comparison of clinical, laboratory and radiological data of studied cases (n = 50) before and 3 months after Zn supplementation

	Before Zn	After Zn	z	p
Height SDS	−3.12 ± 0.2	−1.87 ± 0.1	6.76	0.033*
	(−2.15- -4.21)	(−2.0- -3.11)		
Weight SDS	-1.88 ± 0.62	-1.72 ± 0.51	1.83	0.21
	(-2.01- +0.55)	(-2.05- + 0.66)		
BMI (kg/m2)	16.41 ± 0.56	16.58 ± 0.22	1.59	0.11
	(14.33-18.12)	(14.91-18.42)		
BMI SDS	-1.77 ± 0.41	-1.58 ± 0.32	1.80	0.26
	(-2.10- -0.78)	(-0.91- -1.95)		
Serum Zn (μg/dl)	56.76 ± 7.9	148.25 ± 15.4	8.29	<0.001***
	(37.52-93.11)	(77.55-226.13)		
Peak GH after insulin stimulation (ng/ml)	10.65 ± 2.01	12.10 ± 1.13	2.10	0.081
	(7.61-13.20)	(8.90-14.82)		
Peak GH after clonidine stimulation (ng/ml)	11.14 ± 1.41	12.99 ± 1.22	1.15	0.090
	(6.92-14.11)	(8.90-14.82)		
IGF-1 (ng/ml)	96.72 ± 11.5	177.50 ± 9.06	7.12	<0.010**
	(36.52-115.69)	(42.65-213.66)		
IGF-1 SDS	-2.46 ± 0.11	-0.91 ± 0.43	9.16	<0.010**
	(-3.22- +0.55)	(-2.95 - +1.10)		
IGFBP-3 (ng/ml)	2436.12 ± 392	2856.30 ± 411	5.90	0.042*
	(1195.45-3898.15)	(1341.10-3912.51)		
-BA (years)	3.7 ± 1.05	4.12 ± 1.32	1.25	0.081
	(1.81-9.25)	(2.13-9.81)		
-BA SDS	-1.7 ± 1.14	-1.5 ± 1.21	0.13	0.35
	(-2.44 - +0.22)	(-2.10- + 0.65)		

Results are expressed as mean ± SD and range, *p < 0.05, **p < 0.01, ***p < 0.001, Zn: zinc, SDS: Standard deviation score, BMI: Body mass index, GH: Growth hormone, IGF-1: Insulin like growth factor-1, IGFBP-3: Insulin like growth factor binding protein-3, BA: bone age.

Serum Zn levels rose in all patients but reached normal reference ranges in 32 cases (64 %), serum IGF-1 levels rose in 30 cases (60 %) but reached normal reference ranges in 20 cases (40 %) and serum IGFBP-3 levels rose in 20 cases (40 %) but reached normal reference ranges in 11 cases (22 %). There were no adverse effects other than mild nausea in some patients during the Zn supplementation period.

### Relation between serum Zn and each of laboratory and radiological parameters among studied cases

**Figure 1:**
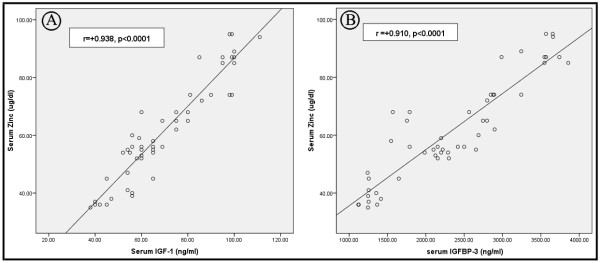
**Correlations between serum zinc and each of serum IGF-1** (Figure 1A) **and serum IGFBP-3** (Figure 1B)**.**

There were significant positive correlations between serum Zn level and each of serum IGF-1 (r = 0.938, p < 0.0001, Figure 1A), IGF-1 SDS (r = 0.81, p < 0.001) and IGFBP-3 levels (p = 0.910, p < 0.0001, Figure 1B) before Zn supplementation. On the other hand, serum Zn did not correlate significantly with peak stimulated GH after insulin induced hypoglycemia (r = 0.17, p = 0.31) and clonidine stimulation (r = 0.13, p = 0.23), BA (r = 0.15, p = 0.13) and BA SDS (r = 0.11, p = 0.22).

## Discussion

Zn deficiency in humans is widespread throughout the world especially in developing countries. In Egypt, growth retardation and short stature due to Zn deficiency are commonly observed. This could be explained by the fact that intakes of animal products and animal protein are very low due to low socio-economic status of the population. In addition, Zn intake is not only low, but its bioavailability is poor because of the high phytate, fiber and tea content of the diet among Egyptian population [15]. Zn deficiency during the period of growth results in growth failure and lack of gonadal development, especially in males. Other effects of Zn deficiency include skin changes, poor appetite, mental lethargy, delayed wound healing, neurosensory disorders and cell-mediated immune disorders [1].

Cesur et al, 2009 [16] found that serum IGF-1 and IGFBP-3 were below normal reference ranges in 96.6 % and 100 % of their short children with Zn deficiency which is close to our findings (94 % and 100 % respectively). It is not known what mechanism is responsible for growth retardation in Zn deficiency and how Zn therapy stimulates growth in children [1]. Moreover, the exact mechanism of the effects of Zn deficiency and Zn supplementation on GH secretion, serum IGF-1 levels, and growth is not well delineated [3].

The current study revealed significant increases in height SDS, GV and its SDS; serum Zn, IGF-1 and IGFBP-3 after 3 months of Zn therapy with no difference in GV between Zn deficient cases and non Zn-deficient controls. Our findings were supported by other studies [3-5,17,18] who reported that Zn supplementation was effective for inducing growth in short children with Zn deficiency in a rate similar to that in healthy children. However, in some studies, this effect was not observed [6,7]. Ninh et al [19] showed that Zn supplementation increased both weight and height after five months compared with placebo treatment but this was not the case in our study concerning the weight. According to these findings, some studies [19-21] suggested that the growth- stimulating effect of Zn might be mediated through changes in circulating IGF-1 levels.

On the other hand, an experimental study in rats [22] showed that restoration of normal circulating levels of IGF-1 and IGFBP-3 by recombinant IGF-1 infusion failed to reverse the growth retardation induced by Zn deficiency. They suggested that growth retardation related to Zn deficiency is caused not only by low serum IGF-1 concentrations, but also by inhibition of the anabolic actions of IGF-1. Moreover, it has been shown that Zn deficiency also leads to alterations in the distribution of serum IGFBPs, in addition to lower circulating IGF-1 concentrations [21]. This theory was further supported by the strong positive correlations between serum Zn and each of IGF-1, IGF-1 SDS and IGFBP-3 levels reported in our study. In addition, other studies [20,21] suggested a state of GH resistance rather than GHD in case of Zn deficiency.

In addition, Imamoglu et al [23] reported that Zn supplementation increased IGF-1 and IGFBP-3 in non-Zn-deficient children with idiopathic short stature. They suggested several explanations for this observation. First, Zn supplementation may have increased the sensitivity to endogenous GH. Secondly, Zn supplementation may have increased the physiological GH secretion (i.e. sleep associated) without altering GH response to pharmacological stimulation. Alternatively, Zn may have non-GH mediated (direct) effects on the synthesis of IGF-1 and IGFBP-3. Support for the first hypothesis comes from animal studies in which ionic Zn exerted a dose dependent stimulation of hGH specific binding to isolated rat adipocytes [24]. Furthermore, Zn ion was found to induce dimerization of human GH [25] and to enhance the bioactivity of hGH [26]. In vivo studies demonstrated that Zn deficiency markedly decreased expression of IGF-1 and GH receptor genes in the liver of growing rats [27]. Support for the third hypothesis comes from the observation that Zn deficiency causes a post-receptor defect in GH action, and a decrease in IGF-1 gene expression which is reversed by Zn supplementation but not by GH administration [28]. This hypothesis was further supported in our study by the finding of higher GV after 3 months of Zn supplementation in GH-deficient than non GH-deficient, both having Zn deficiency.

On the other hand, some reports found increased GH response to pharmacological stimulation after Zn supplementation in Zn-deficient children [5,21,29,30] which was not the case in our study. Siklar et al [30] suggested that during GH treatment in GH deficient children, Zn status should be evaluated, as severe Zn deficiency could decrease the response to GH treatment which is improved with correction of Zn deficiency [30].

In the current study, after 3 months of Zn supplementation, serum Zn levels rose in all patients but reached normal reference ranges in 32 cases (64 %), serum IGF-1 levels rose in 30 cases (60 %) but reached normal reference ranges in 20 cases (40 %) and serum IGFBP-3 levels rose in 20 cases (40 %) but reached normal reference ranges in 11 cases (22 %). Similar results were obtained by Cesur et al, 2009 [16] who confirmed the fact that Zn supplementation affects serum IGF-1 and IGFBP-3 levels in children with Zn deficiency but due to the fact that Zn supplementation was given for only two months, the increases in serum IGF-1 and IGFBP-3 levels were still lower than the normal range in most of their children; therefore, they suggested that Zn supplementation may be necessary for longer periods.

## Conclusions

In conclusion, serum IGF-1 and IGFBP-3 levels were found to be low in short children with Zn deficiency, and increased after Zn supplementation for 3 months but their levels were still lower than the normal reference ranges in most children; therefore, Zn supplementation may be necessary for longer periods. In addition, GV did not differ between Zn-deficient and healthy children with a better GV observed in GH-deficient children. Further studies on larger population scales and for longer periods are warranted to explore the mechanism by which Zn deficiency affects GH- IGF axis and to assess the effect of Zn supplementation on it.

## Competing interests

All authors declare that they do not have any financial or non financial competing interests.

## Authors' contributions

RTH conceived the study, participated in its design and coordination, drafted the manuscript, collected demographical and clinical data of the children and gave final approval of the version to be published. AIH participated in its design and coordination, carried out the laboratory studies and performed the statistical analysis. MTS carried out the laboratory studies and helped in the statistical analysis and drafting of manuscript. All authors read and approved the final manuscript.

## Authors' information

- RTH: Assistant Professor of Pediatrics and Pediatric Endocrinology, Ain Shams University, Cairo, Egypt.

- AIH: Lecturer of Clinical Pathology, Ain Shams University, Cairo, Egypt.

-MTS: Lecturer of Clinical and Chemical Pathology, National Research Center, Cairo, Egypt.
